# Bovine Lactoferrin to Prevent Neonatal Infections in Low-Birth-Weight Newborns in Pakistan: Protocol for a Three-Arm Double-Blind Randomized Controlled Trial

**DOI:** 10.2196/23994

**Published:** 2021-03-11

**Authors:** Shabina Ariff, Sajid Soofi, Almas Aamir, Michelle D'Almeida, Arzina Aziz Ali, Ashraful Alam, Michael Dibley

**Affiliations:** 1 Department of Pediatric and Child Health Aga Khan University Karachi Pakistan; 2 School of Public Health Faculty of Medicine and Health The University of Sydney Sydney Australia

**Keywords:** bovine lactoferrin, low birth weight, sepsis, human milk, premature, mortality

## Abstract

**Background:**

Sepsis is a common and severe complication in premature neonates, particularly those born with low birth weights (<2500 g). Neonatal sepsis is steadily emerging as a leading cause of neonatal mortality in Pakistan. Lactoferrin is a natural product with broad-spectrum antimicrobial properties and glycoprotein that is actively involved in innate immune host responses. Clinical trials have revealed its protective effect on sepsis, but lactoferrin dosage, duration, and role in the prevention of sepsis are still uncertain.

**Objective:**

We aimed to establish the efficacy of bovine lactoferrin in the prevention of late-onset sepsis and to determine the optimal dose and method of administering bovine lactoferrin that may contribute to improvement in overall survival of low birth weight infants.

**Methods:**

We will implement the study in 2 phases at the Aga Khan University Hospital. The first phase, which we have completed, was formative research. This phase mainly focused on a qualitative exploration of perceptions about feeding and caring practices of low birth weight newborns and a trial of improved practices for the preparation and administration of bovine lactoferrin to newborns. The second phase is a 3-arm double-blind randomized controlled trial. In this phase, we randomly allocated 2 different daily oral prophylactic doses of bovine lactoferrin (150 mg or 300 mg) and placebo to 300 low–birth weight neonates starting within the first 72 hours of birth and continuing for the first 28 days of life.

**Results:**

The study protocol was approved by the Ethics Review Committee of Aga Khan University on August 16, 2017. Data collection began in April 2018 and was completed in September 2020. Data analyses are yet to be completed. We expect the results to be published in peer-reviewed journals by autumn of 2021.

**Conclusions:**

This intervention, if effective, has the potential to be translated into a safe, affordable, and widely utilized treatment to prevent sepsis and, subsequently, may improve the survival outcomes of low birth weight neonates in Pakistan and other low- and middle-income countries.

**Trial Registration:**

ClinicalTrials.gov NCT03431558; https://clinicaltrials.gov/ct2/show/NCT03431558

**International Registered Report Identifier (IRRID):**

PRR1-10.2196/23994

## Introduction

Globally, 15.5% of newborns are born with low birth weight, with 96.5% of these births occurring in low-income countries [1]. Approximately 4 million babies in their neonatal period die each year [2]. An estimated 1 million of these deaths occur due to infectious causes, including sepsis, pneumonia, and meningitis, and 60% to 80% of all neonatal deaths are low-birth-weight newborns [1,3]. According to 2018 data, the neonatal mortality rate in Pakistan is 42 per 1000 live births [4]. The current rate of reduction in neonatal mortality is insufficient to achieve United Nations Sustainable Development Goal 3 of reducing neonatal mortality to less than 12 per 1000 live births by 2030 [5].

The incidence of sepsis in neonates is inversely proportional to gestational age and birth weight [6]. Late-onset sepsis is sepsis arising after the perinatal period (>72 hours following birth), which occurs due to microorganisms acquired during the delivery or hospital stay [6,7]. In Pakistan, sepsis caused by multidrug-resistant organisms is common and challenging to treat, resulting in sepsis-related mortality [8]. Late detection, poor sensitivity of diagnostic tests, and nonspecific clinical features further delay the recognition of neonatal sepsis. Furthermore, detection and treatment may not always prevent the high costs of prolonged hospital stays in neonatal intensive care units and late or impaired neurodevelopment. Multiple interventions have been introduced to prevent the morbidity and mortality associated with such serious infections. It is well established that breast milk has beneficial effects against neonatal sepsis in vulnerable populations [9]. This protection is due to the multiple anti-infective, anti-inflammatory, and immunoregulatory factors transmitted through milk. These include secretory antibodies, glycans, lactoferrin, leukocytes, cytokines, and other components produced by the mother’s immune system [9].

Lactoferrin is a whey protein in mammalian milk. It has a multifunctional role in host defense via a myriad of antimicrobial and anti-inflammatory functions. The concentration of lactoferrin is highest in colostrum, decreasing as milk matures, and the decrease is slower in the milk of mothers of preterm infants [10]. Bovine lactoferrin shares similar bioactivity and homology (77%) with its isoform, human lactoferrin, and exhibits even higher in vitro antimicrobial activity than that exhibited by human lactoferrin [11]. A pilot study [12] in the United States found a protective effect of bovine lactoferrin–enhanced formula (containing 850 mg bovine lactoferrin/L) against lower respiratory tract illnesses in infants compared to that of regular formula. A recent review [13] concluded that lactoferrin was a safe intervention, and its most likely future use in children would be to protect against enteric infections and neonatal sepsis. Furthermore, it concluded that bovine lactoferrin had the potential in to reduce sepsis in low-birth-weight infants and aid in the reduction of child mortality [13]. A randomized clinical trial [14] in India to evaluate the efficacy of bovine lactoferrin in preventing late-onset sepsis in low-birth-weight neonates reported a significant reduction in the incidence of late-onset sepsis in the bovine lactoferrin supplementation group compared to the placebo.

Despite promising experimental data, clinical information on bovine lactoferrin in infants is scarce, with minimal data from low- and middle-income countries [14]. We sought to address the gaps in the current literature regarding the optimal dose, administration method, and efficacy of bovine lactoferrin in decreasing the incidences of late-onset sepsis and necrotizing enterocolitis in low-birth-weight infants in Pakistan. We also aim to determine the role of bovine lactoferrin supplementation in improving neonatal survival.

The primary objective of this study is to evaluate bovine lactoferrin efficacy in reducing the incidence of late-onset sepsis and to define the optimal daily dose and administration method of bovine lactoferrin to prevent late-onset neonatal sepsis in low-birth-weight and preterm neonates. The secondary objective of this study is to evaluate the effectiveness of bovine lactoferrin in reducing the incidence of necrotizing enterocolitis in low-birth-weight and preterm neonates, and the effectiveness of bovine lactoferrin in improving neonatal survival during the first 28 days of life.

## Methods

### Study Design

This study consists of 2 phases. The first phase was a qualitative component with formative research and a trial of improved practices. The second phase was a 3-arm double-blind individually randomized controlled trial.

In the first phase, we conducted formative research before the initiation of the clinical trial to explore the acceptability of the delivery of the intervention. We used qualitative research methods, such as in-depth interviews, focus group discussions, and direct observations at the household level, to ascertain the practices and perceptions around feeding and care of low-birth-weight newborns. Based on the information from themes related to adherence, feasibility, and acceptability of bovine lactoferrin use, we carried out an additional trial of improved practices of preparation and administering of bovine lactoferrin to the low-birth-weight newborn by the caregivers at home.

In the second phase, we conducted a 3-arm randomized controlled trial to compare 2 different prophylactic oral doses of bovine lactoferrin (150 mg or 300 mg) and placebo, given daily over the first 28 days of life. We initiated the treatment within the first 72 hours following birth.

### Study Setting

This study was conducted at the Aga Khan University Hospital in Karachi, Pakistan, in collaboration with researchers from the School of Public Health, the University of Sydney, Australia. The Aga Khan University Hospital is a state-of-the-art Joint Commission International accredited tertiary care hospital and has 2 postnatal wards with a fully equipped 24-bed neonatal intensive care unit with an extensive clinical support unit. Annually, approximately 5500 deliveries take place with a known prevalence of 15% to 18% for low-birth-weight neonates. The hospital also has a clinical trial unit, laboratory, and pharmacy, along with other essential hospital services to conduct clinical trials.

### Study Population and Eligibility Criteria

For the formative research, we recruited women, their husbands, and mothers or mothers-in-law from Aga Khan University Hospital, and the qualitative researcher made an appointment to interview them at the hospital using a standard structured questionnaire. The trial participants, parents with low-birth-weight neonates (birth weight <2500 g and ≥ 1000 g) in each treatment group, were recruited from the Aga Khan University Hospital. All low-birth-weight neonates with gestational age from 28 weeks to 36 weeks 5 days, with no sign of sepsis, and on oral feeding within 72 hours of birth were eligible. We obtained written informed consent from parents or caregivers after assurance that the family planned to stay in the study area for at least 1 month.

Exclusion criteria were neonates with congenital anomalies, culture-proven early-onset sepsis, history of severe maternal chorioamnionitis, or group B streptococcus colonization during pregnancy, gestational age less than 27 weeks 6 days, birth weight less than 1000 g, and parental refusal to consent.

### Sample Size

#### Phase 1: Formative Research

Following the inclusion criteria, 10-15 low-birth-weight neonates’ parents or caregivers were recruited purposively for the trial of improved practices. We conducted 30 in-depth interviews.

#### Phase 2: Clinical Trial

A total of 100 low-birth-weight neonates were enrolled in each group, leading to an aggregate of 300 neonates. We estimate that the sample size with a 2-sided α of 5%, power of 80%, and 10% lost to follow up. Based on current hospital data, the incidence of neonatal sepsis in the placebo group is 25%. We conducted 30 in-depth interviews with parents and caregivers of low-birth-weight infants. We enrolled 15 low-birth-weight infants for trial of improved practices. Based on these assumptions, the required sample size in each trial arm was calculated as 95 low-birth-weight neonates (rounded to 100 low-birth-weight neonates), leading to a total of 300 low-birth-weight neonates.

### Randomization, Allocation, and Masking

An independent statistician from the University of Sydney produced a computer-generated random allocation sequence using Stata (version 15; StataCorp LLC) software with a 1:1:1 ratio for the 3 arms of the trial (an allocation probability of one-third per treatment arm) and a block size of 9 for approximately anticipated number of newborns recruited each day.

Once an infant met the eligibility criteria, we provided an information sheet to the parents, in English and the local language, explaining (1) the benefits of lactoferrin as a nutritional supplement along with the method of administration and (2) the placebo and its ingredients. Informed consent was obtained from the parents, after which we enrolled the neonates in the study. We blinded the participants and study teams to the group allocation. We delivered the treatment kits to the study site before initiation of the study.

### Intervention

We will evaluate 2 different strengths (150 mg and 300 mg) of bovine lactoferrin as the intervention treatment. The color of bovine lactoferrin powder in each intervention and placebo (D–Glucose) are identical in appearance. The study staff administered the first oral dose of the trial supplement to the neonate at the hospital in the presence of the mother or the caregiver on the third day of life with a single daily dose mixed with milk (preferentially breast milk otherwise formula milk) and the mother or caregiver continued to administer the supplement/placebo for 28 days. We stored the supplement sachets at room temperature and maintained an inventory at the clinical trial unit at the Aga Khan University Hospital. We monitored adherence by weekly collection of used empty sachets from the parents before providing them with the next set of sachets for the subsequent week.

### Retention and Follow-up

We followed the newborns weekly (for 28 ± 2 days) for general well-being, recognition of danger signs, and adherence to supplementation. Daily follow-ups were completed during the hospital stay until discharge. At the time of discharge, we provided the participants with 1 week’s supply of supplements. A telephone follow-up was conducted midweek, and a home visit was conducted once a week. The study investigators conducted the first follow-up visit after discharge from the neonatal care unit.

### Data Collection and Management

The research team captured data electronically using tablets equipped with special-purpose programs. We ran a pilot before the implementation of the trial to avoid technical errors. The tablets transferred the captured data to a secured database on the server at Aga Khan University with a secure copy at the University of Sydney.

At each follow-up visit, at home or at the hospital, a questionnaire was administered about the history of illnesses and feeding practices during the previous week. The criteria for identifying a baby with presumed clinical sepsis included any signs or symptoms such as lethargy, apnea, tachypnea, respiratory distress, reluctance to feed, temperature instability, feed intolerance, vomiting, aspirates, abdominal distention, and seizures. We define sepsis as a baby with any of the criteria of presumed sepsis in addition to either increased C-reactive protein level (>1 mg/dL) or an increased or decreased white blood cell count (7.0-23.0×10^3^/uL), hypoglycemia (<40 mg/dL), or positive blood culture.

We conducted a complete physical examination on every visit. In addition, anthropometric measurements were taken at birth, on day 14, and on day 28. If any of the above symptoms were found or there was clinical suspicion of sepsis in a neonate, we performed a workup (sepsis screen) according to the standard protocol of the hospital, which includes complete blood count, C-reactive protein level, blood culture, and admission.

### Statistical Analysis

We will use SPSS software (version 19.0; IBM Corp) for statistical analysis. For continuous data, we will report the mean and standard deviation or median and interquartile range. We will report the baseline characteristics of the study population with frequencies and percentages. To compare categorical variables, we will use chi-square tests, and to compare continuous variables, we will use paired *t* tests. To assess the outcome variable, incident neonatal sepsis, we will use applied logistic regression and report odds ratios and 95% confidence intervals for all predictor variables. Initially, we will conduct bivariate logistic regression analyses to estimate an unadjusted odds ratio for each variable. We will examine the predictors that demonstrate an association with the outcome (*P* value <.25) in multiple logistic regression models. We will consider a *P* value ≤.05 as significant.

### Safety Reporting

Previously published evidence shows that orally administered lactoferrin decreases sepsis and necrotizing enterocolitis in preterm neonates without any adverse effects [15,16]; however, if any adverse events occur, it will be reported in accordance with local institutional ethics committee policies. We will define an adverse event as any untoward medical occurrence in a trial participant who has been administered the trial product and which does not necessarily have a causal relationship with the treatment.

Serious adverse events will be any untoward medical occurrence that at any dose results in death, is life-threatening (ie, the infant is at risk of death at the time of the event), requires inpatient hospitalization or prolongation of existing hospitalization, or other important medical events which, in the opinion of the investigator, are likely to become serious if untreated.

A suspected unexpected serious adverse reaction will be defined as an serious adverse event that is related to the drug or device and is unexpected (ie, not listed in the investigator brochure or approved Product Information and Informed Consent Form [17]).

### Ethical Approval and Consent to Participate

The Ethics Review Committee of the Aga Khan University approved the study (ID 4873-Ped-ERC-17). The National Bioethics Committee of Pakistan granted approval for the study to be conducted on human participants. The study was also approved by the Research Integrity & Ethics Administration, Human Research Ethics Committee, The University of Sydney (ID 2017/420).

Written informed consent was obtained from all study participants prior to enrollment in the study. We will take special care to ensure the confidentiality and privacy of the study participants’ medical histories and personal information. We will store all sources of information in a password-protected predefined program, using tablets (at the time of enrollment and subsequent follow-up visits) at the Data Management Unit (The Aga Khan University). All source documents will be maintained in locked files and will only be available to people directly involved in the study.

## Results

The study protocol was approved on August 16, 2017. Data collection began in April 2018 and was completed in September 2020. Data analyses are yet to be completed. We expect the results to be published in peer-reviewed journals by autumn 2021.

## Discussion

This study will provide information on the effectiveness of bovine lactoferrin in preventing late-onset neonatal sepsis in low-birth-weight neonates and will be used to identify the optimal dosage and administration method. Our study approach aims to prevent neonatal infections; current treatment approaches involve treating neonatal infections when they occur. The current approach, which focuses on early detection of infections in newborns through postnatal care and treatment with antibiotics, runs the risk of worsening antimicrobial resistance, which is already an emerging crisis. Our approach, through the use of prophylactic bovine lactoferrin supplements, will be cost-effective, greatly reduce the risk of microbial resistance to antibiotics, and can be implemented in all settings through various health care providers who would initiate the treatment.

A review [15] of clinical trials found that despite the small sample size, the preliminary results of the trials showed a positive effect of lactoferrin on the prevention of neonatal infections. It also concluded that if proven to be effective, lactoferrin can become a cost-effective intervention that can potentially impact neonatal mortality and morbidity worldwide [15]. In a 2017 Cochrane review [16], enteral lactoferrin was found to reduce the rate of late-onset sepsis (risk ratio 0.59, 95% CI 0.40-0.87; *P*=.008; from 6 trials with 886 participants) and the rate of necrotizing enterocolitis (risk ratio 0.40, 0.18-0.86; *P*=.02; from 4 trials with 750 participants) in preterm neonates. The review [16] also concluded that although lactoferrin holds great potential for late-onset sepsis and necrotizing enterocolitis prevention, multiple questions still need to be answered, including the administration method and optimal dosage. Our study aims to address these gaps in the current literature.

This intervention, if effective, has the potential to be translated into a safe, affordable, and widely utilized intervention to prevent sepsis and subsequently improve survival in low-birth-weight neonates in Pakistan and other low-and middle-income countries. Following the achievement of conclusive results, we plan to scale up the intervention. Our long-term vision is to produce lactoferrin within Pakistan as a byproduct of the dairy industry, for use as part of the standard neonatal care package for the support of low-birth-weight babies, delivered both in hospital and at home.

The results of this trial will be disseminated to the government, academic, and policy-making communities, as well as to the wider public in a peer-reviewed journal and at relevant national and international conferences.

## References

[1] Care of the preterm and low-birth-weight newborn. World Health Organization.

[2] Lawn JE, Rudan I, Rubens C (2008). Four million newborn deaths: is the global research agenda evidence-based?. Early Hum Dev.

[3] Lawn JE, Cousens S, Zupan J, Lancet Neonatal Survival Steering Team (2005). 4 million neonatal deaths: when? where? why?. Lancet.

[4] Mortality rate, neonatal (per 1,000 live births). The World Bank.

[5] Goal 3: ensure healthy lives and promote well-being for all at all ages. United Nations Sustainable Development Goals.

[6] Kaufman D, Fairchild KD (2004). Clinical microbiology of bacterial and fungal sepsis in very-low-birth-weight infants. Clin Microbiol Rev.

[7] Hornik CP, Fort P, Clark RH, Watt K, Benjamin DK, Smith PB, Manzoni P, Jacqz-Aigrain E, Kaguelidou F, Cohen-Wolkowiez M (2012). Early and late onset sepsis in very-low-birth-weight infants from a large group of neonatal intensive care units. Early Hum Dev.

[8] Rahman S, Hameed A, Roghani MT, Ullah Z (2002). Multidrug resistant neonatal sepsis in Peshawar, Pakistan. Arch Dis Child Fetal Neonatal Ed.

[9] Furman L, Taylor G, Minich N, Hack M (2003). The effect of maternal milk on neonatal morbidity of very low-birth-weight infants. Arch Pediatr Adolesc Med.

[10] Hirai Y, Kawakata N, Satoh K, Ikeda Y, Hisayasu S, Orimo H, Yoshino Y (1990). Concentrations of lactoferrin and iron in human milk at different stages of lactation. J Nutr Sci Vitaminol (Tokyo).

[11] Buccigrossi V, de Marco G, Bruzzese E, Ombrato L, Bracale I, Polito G, Guarino A (2007). Lactoferrin induces concentration-dependent functional modulation of intestinal proliferation and differentiation. Pediatr Res.

[12] King JC, Cummings GE, Guo N, Trivedi L, Readmond BX, Keane V, Feigelman S, de Waard R (2007). A double-blind, placebo-controlled, pilot study of bovine lactoferrin supplementation in bottle-fed infants. J Pediatr Gastroenterol Nutr.

[13] Ochoa TJ, Pezo A, Cruz K, Chea-Woo E, Cleary TG (2012). Clinical studies of lactoferrin in children. Biochem Cell Biol.

[14] Kaur G, Gathwala G (2015). Efficacy of bovine lactoferrin supplementation in preventing late-onset sepsis in low birth weight neonates: a randomized placebo-controlled clinical trial. J Trop Pediatr.

[15] Turin CG, Zea-Vera A, Pezo A, Cruz K, Zegarra J, Bellomo S, Cam L, Llanos R, Castañeda Anne, Tucto L, Ochoa TJ, NEOLACTO Research Group (2014). Lactoferrin for prevention of neonatal sepsis. Biometals.

[16] Pammi M, Suresh G (2017). Enteral lactoferrin supplementation for prevention of sepsis and necrotizing enterocolitis in preterm infants. Cochrane Database Syst Rev.

[17] (2012). Safety reporting requirements for INDs (investigational new drug applications) and BA/BE (bioavailability/bioequivalence) studies. United States Food and Drug Administration.

